# Non-invasive breath testing to detect colorectal cancer: protocol for a multicentre, case–control development and validation study (COBRA2 study)

**DOI:** 10.1186/s12885-025-14520-2

**Published:** 2025-07-29

**Authors:** Michael G. Fadel, James Murray, Georgia Woodfield, Ilaria Belluomo, Ivan Laponogov, Aaron Parker, Valerio Converso, James K. Ellis, Pete Wheatstone, Julie Hepburn, Chris Groves, Kevin Monahan, Brian P. Saunders, Patrik Španěl, Kirill Veselkov, Amanda J. Cross, Christos Kontovounisios, Linda D. Sharples, George B. Hanna, COBRA2 Collaborators, Natalia Klimowska-Nassar, Melody Ni, Claudia Adade, James Emerton, Stephanie Ivie, Ella Jameson, Lija Joy, Olumayowa Ogunwemimo, Anukriti Panda, Kerry Richards, Elisa Speranzini, Adebayo Tofiat, Metod Oblak, Ethel Black, Karmina Claros, Holly Hogan, Sean Connarty, Parvathy Oby Gopi, Valentina Raspa, Julian Ashton, Annabel Dawson, Maurice Hoffman, Monica Jefford, Carrol Lamouline, Debra Smith

**Affiliations:** 1 Department of Surgery and Cancer, Hammersmith Hospital Campus, Imperial College London, Du Cane Road, London, W12 0NN UK; 2 Department of Medical Statistics, London School of Hygiene and Tropical Medicine, London, UK; 3 Patient and Public Involvement, Clinical Trials Research Unit, University of Leeds, Leeds, UK; 4 Patient and Public Involvement, Health and Care Research Wales Support Centre, Cardiff, UK; 5 Department of Gastroenterology, St George’s Hospital, London, UK; 6 Department of Gastroenterology, St Mark’s Hospital and Academic Institute, London, UK; 7 Department of Chemistry of Ions in Gaseous Phase, J. Heyrovsky Institute of Physical Chemistry of the Czech Academy of Sciences, Prague, Czech Republic; 8 Department of Colorectal Surgery, Chelsea and Westminster Hospital NHS Foundation Trust, London, UK; 9 Department of Colorectal Surgery, Royal Marsden NHS Foundation Trust, London, UK; 10 2nd Surgical Department, Evaggelismos Athens General Hospital, Athens, Greece

**Keywords:** Colorectal cancer, Breath test, Volatile organic compounds, Biomarkers, Detection, Diagnostic model

## Abstract

**Background:**

Colorectal cancer (CRC) is the fourth most common cancer in the United Kingdom. The five-year survival rate from CRC is only 10% when discovered at a late stage, but can exceed 90% if diagnosed early. Symptoms related to CRC can be non-specific, and therefore the decision to refer for a colonoscopy can be challenging. Breath analysis potentially offers a simple and quick method to detect CRC specific volatile organic compounds (VOCs) in breath. This protocol describes the COBRA2 study which aims to develop and validate the clinical prediction model (CPM) in the detection of CRC based on the breath test. An exploratory comparison between the breath test and faecal immunochemical test (FIT) will also be carried out to assess whether combining both tests improves diagnostic performance.

**Methods:**

The COBRA2 study is a multicentre, case-control development and validation study. Breath samples will be collected from participants attending hospital for a planned colonoscopy (control group) or from participants with histologically confirmed colorectal adenocarcinoma (CRC group). A total of 720 participants (470 controls, 250 CRC) will be recruited. All participants will maintain a clear fluid diet for a minimum of 4–6 h prior to sampling, which will take place at outpatient clinics to avoid bowel preparation. The FIT result will be recorded where available. Breath samples will be analysed using gas chromatography–mass spectrometry to identify the VOCs present. Relationships between VOCs of interest and presence of CRC will be explored, and the CPM will be developed using statistical and machine learning methods. We will also assess whether incorporating FIT into the CPM improves diagnostic performance. The CPM will be subsequently validated in an independent sample of up to 250 participants (125 controls, 125 CRC) using the same case–control design and the potential clinical utility of decision rules for triaging will be assessed. If successful, broad validation in an unselected target population of symptomatic patients is required.

**Discussion:**

The non-invasive breath test may provide direct patient benefit through earlier and accurate detection of CRC, and higher patient acceptability. It can help ensure timely secondary care referral, potentially translating to improved curative treatment and survival for patients.

**Trial registration:**

The study is registered with ClinicalTrials.gov (NCT05844514).

**Supplementary Information:**

The online version contains supplementary material available at 10.1186/s12885-025-14520-2.

## Background

In the United Kingdom (UK), colorectal cancer (CRC) is the second most common cause of cancer death, with survival rates amongst the lowest in Europe [1]. If diagnosed early at stage one, the five-year survival is over 90% [2]. However, 23% of patients are diagnosed with advanced disease (stage four) which has a five-year survival of only 10% [3]. Most CRC patients present via the suspected lower gastrointestinal (GI) cancer (30%) or routine (34%) referral pathways, the latter often being associated with significant delay. Twenty-four percent of patients present as an emergency, typically with advanced disease [4].

Early-stage CRC shares many symptoms with common benign conditions and so it can be unclear which patients should be referred for a colonoscopy. Referring all symptomatic patients would overwhelm available resources with demand expected to continue to rise further over the next five years. Annually 1.43 million lower GI endoscopies are performed in the UK, but the diagnostic yield for CRC is lower than 3% [5, 6]. The annual cost of lower Gl endoscopies to the National Health Service (NHS) is estimated to be over £530 million [7]. To improve precision in the early detection of CRC, it is essential to refer patients for colonoscopy who are most likely to have CRC, while not subjecting those at lower risk to an invasive and costly procedure. Therefore, we need sensitive diagnostic tools in primary care that are accurate, easy to perform and accepted by patients.

In the UK, guidelines including the National Institute for Health and Care Excellence (NICE), have recommended the use of the faecal immunochemical test (FIT), which measures the amount of haemoglobin in a stool sample, to triage low-risk symptoms in primary care [8]. Faecal haemoglobin concentrations are known to vary by age, sex, ethnicity, deprivation, cancer stage and iron-deficiency anaemia [9]. Depending on the selected threshold for positivity, sensitivity of FIT for CRC varies between 54 and 93%, specificity between 83 and 95%, with a positive predictive value of 7% in patients with low-risk symptoms [10].

Breath testing is based on the detection of volatile organic compounds (VOCs). VOCs are carbon-containing molecules that are sufficiently volatile to be detectable in a gas form at room temperature [11]. We now know that endogenous VOCs exist as the by- or end-product of biochemical reactions and metabolic processes taking place inside all humans, both healthy and diseased [12]. The non-invasive breath test has the ideal characteristics for a primary care triage tool as it is simple and acceptable to patients of all ethnicities and socioeconomic groups [13], thereby helping to promote equity of healthcare access and optimise patient compliance. There is the opportunity to combine tests based on VOCs with FIT to further enhance performance [14]. In addition, the breath test has the potential as a platform technology to identify other malignant diseases, such as oesophageal, gastric and pancreatic cancers, with different VOC signatures [15, 16]. A primary care clinician faced with non-specific GI symptoms could therefore test for multiple GI cancers from a single breath sample.

Currently, there is limited published data which has assessed the performance of breath VOCs in the detection of CRC [17–23]. In these studies, the performance of VOC-based tests was similar to those of existing stool-based immunohistochemical and DNA tests [24]. However, the potential impact of these studies was limited by sample size, unstandardised methods lacking validation and the non-human origin of some reported VOC markers. In 2022, we completed a discovery study, COlorectal BReath Analysis 1 (COBRA1), which showed the proposed breath test has the potential to detect CRC [25]. A diagnostic model to detect CRC was developed from 855 symptomatic patients (709 controls, 146 CRC) with an area under the receiver operating characteristic (AUROC) curve (± standard error) of 0.91 ± 0.01, sensitivity of 83 ± 2%, specificity of 88 ± 1% and negative predictive value of 96%. This was based on a panel of 14 VOC biomarkers (along with body mass index) identified for CRC. COBRA1 had some limitations that should be acknowledged. This includes heterogeneity of populations and variable use of colonoscopy bowel preparation amongst participants included in the study (although this was not found to be a predictive feature or confounding factor in the VOC-based model). Participants were not sampled if they were receiving any concurrent chemotherapy, but they were not excluded if they had any prior chemotherapy, radiotherapy, or immunotherapy. In addition, the technology was not available at the time to perform robust structural and chemical validation of the VOCs using two-dimensional gas chromatography.

### COBRA2 study objectives

The objectives of the COBRA2 study are to develop and validate a clinical prediction model (CPM), using an improved technology and methodology, to triage the risk of CRC based on profiles of VOCs obtained from the breath test. The clinical utility of the model for referral for the appropriate reference test will be investigated and compared against the current protocol of referring all patients presenting with symptoms indicative of CRC. We will also perform an exploratory comparison between the breath test and FIT, and assess the performance of combining both tests to detect CRC. This will be followed by a narrow validation study using the same case–control design, to assess the ability of the CPM to distinguish between CRC and non-cancer, and to assess the potential clinical utility of decision rules when used for triaging.

We anticipate an improvement in biomarker identification and quantification due to the use of: (i) updated breath collection device and time-of-flight (TOF) and two-dimensional gas chromatography-mass spectrometry (GC–MS) technology; (ii) calibration curves for target compounds; (iii) avoidance of colonoscopy bowel preparation as a possible confounder, and (iv) additional quality control (QC) steps for biomonitoring thermal desorption (TD) tubes, GC-TOF–MS analysis, and a dedicated laboratory environment, therefore enhancing the lower limits of VOC quantification.

## Methods

### Study design

COBRA2 is a multicentre, case–control development and validation study. The study involves collection of breath at a single time point from eligible participants and is open to all NHS trusts. The COBRA2 study is sponsored by Imperial College London and has obtained NIHR Clinical Research Network portfolio adoption to support study recruitment. The COBRA2 study commenced in September 2022 and is due to complete by December 2026.

The following groups of participants are being recruited:

i) Control group: symptomatic patients who are attending a planned colonoscopy referred under the suspected lower GI cancer pathway. Any patient who is found to have histologically-proven CRC on colonoscopy will be analysed as part of the CRC group.
ii) CRC group: patients who either have a confirmed diagnosis of colorectal adenocarcinoma according to a biopsy, or who are due to undergo surgical resection for suspected CRC (with histological confirmation to follow within three months).

The target recruitment for the development of the CPM is 720 patients (470 controls, 250 CRC), aiming for a total of 576 patients (376 controls, 200 CRC) with reliable and complete data (breath test and reference test). For the narrow validation, the target recruitment is up to 250 patients (125 controls, 125 CRC), aiming for 200 patients (100 controls, 100 CRC) with reliable and complete data.

### Participant eligibility

The following participants are eligible for inclusion:

- Patients aged ≥ 18 years referred from primary care with symptoms of suspected CRC.
- Patients with histologically confirmed colorectal adenocarcinoma (stages I-IV) who are treatment naïve (CRC group).

Participants with any of the following will not be eligible for inclusion:

i) Previous surgery altering the anatomy of the lower GI tract (e.g., hemicolectomy, anterior resection).
ii) Previous treatment (neoadjuvant chemotherapy or radiotherapy or immunotherapy) for CRC.
iii) Received bowel preparation for their colonoscopy procedure.
iv) History of any other cancer within three years.
v) Unable or unwilling to provide informed written consent.

### Sample size

We have powered our study on sensitivity given the poor prognosis of CRC and the clinical importance of minimising missed diagnoses. The sample size calculation aimed to estimate sensitivity and specificity to acceptable precision using a one-sided Binomial test against a fixed target sensitivity. This was implemented using the R package pwr [26]. Sample size was explored for target sensitivities of 85% to 95% and specificities of 80% to 90%, based on the results from the COBRA1 study. For a target sensitivity of 90%, a sample of 200 CRC cases yields 90% power for the lower (one-sided) 95% confidence interval (CI) limit to be ≥ 83%. For a target specificity of 85%, based on the published specificity for FIT at 10 µg/g, a sample size of 262 controls provides 90% power for the lower limit of the 95% CI to be ≥ 78%. Additional controls (total 376) are included to allow assessment of heterogeneity. Reliable and complete data is therefore required in 576 patients (376 controls, 200 CRC). To account for 20% attrition (e.g., incomplete colonoscopy or breath data), we will recruit a total of 720 patients (470 controls and 250 CRC cases) for CPM development. This sample size allows us to estimate the AUROC with a standard error of less than 2% provided it is at least 0.8, and provides acceptable power for estimating other performance measures. Sample size estimation is consistent with methods suggested by Riley et al. [27] for development of diagnostic models. In the narrow validation study, we aim to recruit an equal number of CRC cases and controls, and similarly allow for a 20% attrition rate. The calculated sample size of up to 250 patients (125 controls, 125 CRC), based on the target sensitivity and specificity (85% to 90%), will ensure sufficient precision in estimating diagnostic accuracy (standard errors for sensitivity and specificity 3.5% to 5%), discrimination (standard error for AUROC < 3%), and calibration (standard error for observed/expected cases < 7.5%), whilst maintaining feasibility [28].

### Participant enrolment

**Fig. 1:**
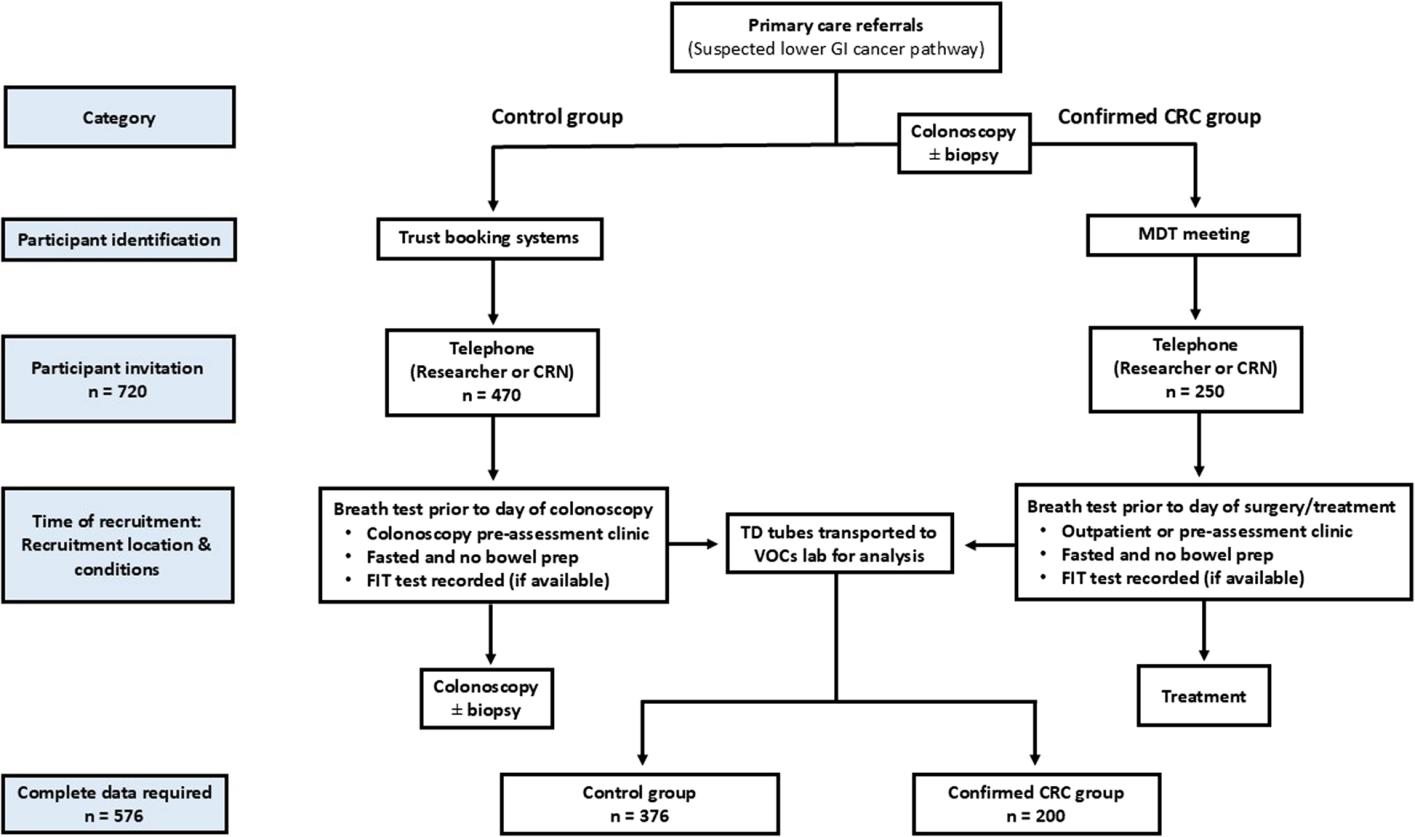
Recruitment pathway for breath testing in control and colorectal cancer cases. Abbreviations: CRC, colorectal cancer; CRN, Clinical Research Network; FIT, faecal immunochemical test; MDT, multidisciplinary team; TD, thermal desorption; VOCs, volatile organic compounds

Participants will be identified from suspected lower GI cancer pathway from NHS trust booking systems (control group) and multidisciplinary team meetings (CRC group) (Fig. 1). Participant eligibility will be confirmed, and participants will be contacted by research staff by telephone at least 24–48 h prior to breath testing. Study details will be provided, including the need to maintain a clear fluid diet for a minimum of 4–6 h prior to testing. The participant information leaflet will be sent to the patient via email or post. Informed consent will be taken by the research staff at the scheduled hospital attendance. Breath sampling will take place at outpatient or pre-assessment clinics for controls and CRC cases prior to any endoscopy or surgery. These in-person endoscopy and surgery pre-assessment appointments are part of patients’ routine care in the study hospitals and therefore patients usually do not need to make additional visits to the hospital for testing. Neither groups will have had bowel preparation prior to their research visit. Using a structured proforma to allow integration of symptoms in the diagnostic model, patient demographics, co-morbidities, medication history, symptoms, cancer characteristics and FIT results are being recorded from either primary or secondary care records (Supplementary material Table S1).

### Breath sample collection

Participants will be asked to provide a breath sample (up to 2 L) by exhaling directly into a single-use breath collection bag via a mouthpiece that is subsequently sealed. A breath sampling system will pump breath from the bag onto two TD tubes, with a double-bed sorbent phase composed of Tenax TA/Carbograph 5TD (Markes International Ltd, Llantrisant, UK), within an air-tight system at a controlled flow rate of 200 ml/min [29]. This process will be repeated, and the room air will then be sampled using two further TD tubes to control for potential background environmental contamination. The tubes will be immediately sealed with airtight brass caps and transported to the Imperial VOC laboratory for same day mass spectrometry analysis. When analysis is not possible within 48 h, TD tubes will be dry purged prior to storage at −80 °C for subsequent analysis.

### Volatile organic compound analysis

Breath and room air samples will be analysed, with established Standard Operating Procedures at Imperial College London, using TD-GC-ToF–MS instruments (Markes TD100-XR-Agilent 6890-BenchTOF Select, SepSolve, UK) equipped with polar and mid-polar columns. Two TD tubes will be analysed using both mid-polar and polar GC-ToF–MS. VOCs will be recollected and analysed using a two-dimensional TD-GCxGC-ToF–MS (TD100-XR-Agilent 7890-Sepsolve modulator-BenchTOF Select-eV, Sepsolve, UK) for robust structural identification of VOCs. The acquired spectra will be compared against the National Institute of Standards and Technology (NIST) mass spectral library and authentic standards will be then used for chemical verification of compounds. Data will be extracted, pre-processed and quality controlled using ChromSpace (Markes, UK) and in-house scripts. The VOCs discovered during this analysis will also be compared with previously identified VOCs from the COBRA1 study. All study data will be collected according to the Data Protection Act 2018 and in line with General Data Protection Regulation (GDPR).

### Quality control

QC mechanisms in our laboratory include: (i) coding of TD tube usage and background level check prior to tube transport to site; (ii) transport of tubes on a weekly basis via dedicated couriers using standardised inert transport containers; (iii) upon arrival to laboratory, tubes are barcoded along with anonymised patient ID to ensure full traceability; (iv) tube storage in dedicated prenumbered slots to facilitate sample identification pre-analysis; and (v) electronic audit trail of tube use within a secure Laboratory Information Management System (LIMS). Breath sample analysis includes (i) daily QC of TD-GC-ToF–MS instruments to evaluate the precision of measure of VOCs within the run and in-between runs; (ii) ensuring the presence of quality breath sample using a threshold for a reference endogenous compound; (iii) coding and linking mass spectra that pass QC standards to metadata; and (iv) automated high throughput system.

### Statistical analysis

The COBRA2 study is a development and narrow validation study which follows the validation strategy described by Cowley et al. [30] and Transparent Reporting of a multivariable prediction model for Individual Prognosis Or Diagnosis (TRIPOD) guidelines [31]. This method ensures the development of a rigorous and robust CPM that maximises the potential benefit on patient care and decision-making in clinical practice. For development and validation, we use a case–control design for efficiency given that CRC has a low prevalence in the population. Patients referred on the suspected lower GI cancer pathway have a CRC risk of approximately 5–7% [32]. We will use statistical methods for model development and validation. Initially, we will confirm previously identified VOCs using enhanced measurement methods. Using VOCs from COBRA1 as a base, a screening step will identify the most promising additional predictors. Then logistic regression methods will be used to develop a model based on the breath test alone, including all identified VOCs. To reduce over-fitting, we will use model reduction methods such as the elastic net, least absolute shrinkage and selection operator (LASSO) or Harrell’s stepdown approach [33]. For internal model validation, the following will be used [33, 34]:

1. 10-fold cross-validation and median values of the performance measures across folds.
2. For calibration, predicted risks of CRC will be plotted against the observed risks.
3. For overall predictive performance Mean Absolute Prediction Error will be estimated (0%: perfect prediction, 100%: no predictive value).
4. For discrimination, the non-parametric AUROC will be calculated, and the threshold that achieves the best trade-off between sensitivity and specificity will be extracted.

For final models, we will use logistic regression modelling to also include patient and clinical characteristics. We will assess linearity of relationships between CRC risk and continuous variables, and explore categorical variables and two-way interactions, including interactions with the breath test. All candidate predictors will initially be entered into the model, before simplification based on Wald statistics (significance < 0.05), size of the predictive weight (odds ratio) and clinical opinion. If there are missing patient or clinical characteristics, we will consider using imputation techniques [35].

In addition to the statistical methods, random forest and deep neural networks (DNNs) will be explored to assess whether they can improve model predictions. Random forest will be used to construct multiple decision trees from data subsets and combine their predictions to identify key VOCs that predict CRC. DNNs will be employed to model nonlinear relationships in the VOC data. Data analysis and data cleaning will be carried out using the statistical computing language R [36]. We will also calculate the sensitivity, specificity and AUROC of the FIT results (where available). We will compare these quantities informally since there will not be sufficient power for a statistical comparison. We will then assess whether incorporating FIT into the final CPM improves diagnostic performance.

### Data monitoring

The non-invasive breath test exposes participants to minimal risk. All adverse events considered to be related to the collection of the breath samples will be reported to the COBRA2 trial management group via the adverse event reporting form (as a paper copy or on the REDCap study database). Serious adverse events will be reported to the trial management group within 24 h of the site becoming aware of the event, and will be reported to the Sponsor and Research Ethics Committee, as required by the Standard Operating Procedures of the Imperial College London Research Governance and Integrity Team. The trial management group will monitor recruitment, treatment and attrition rates, and any concerns related to the study.

### Patient and public involvement

**Fig. 2:**
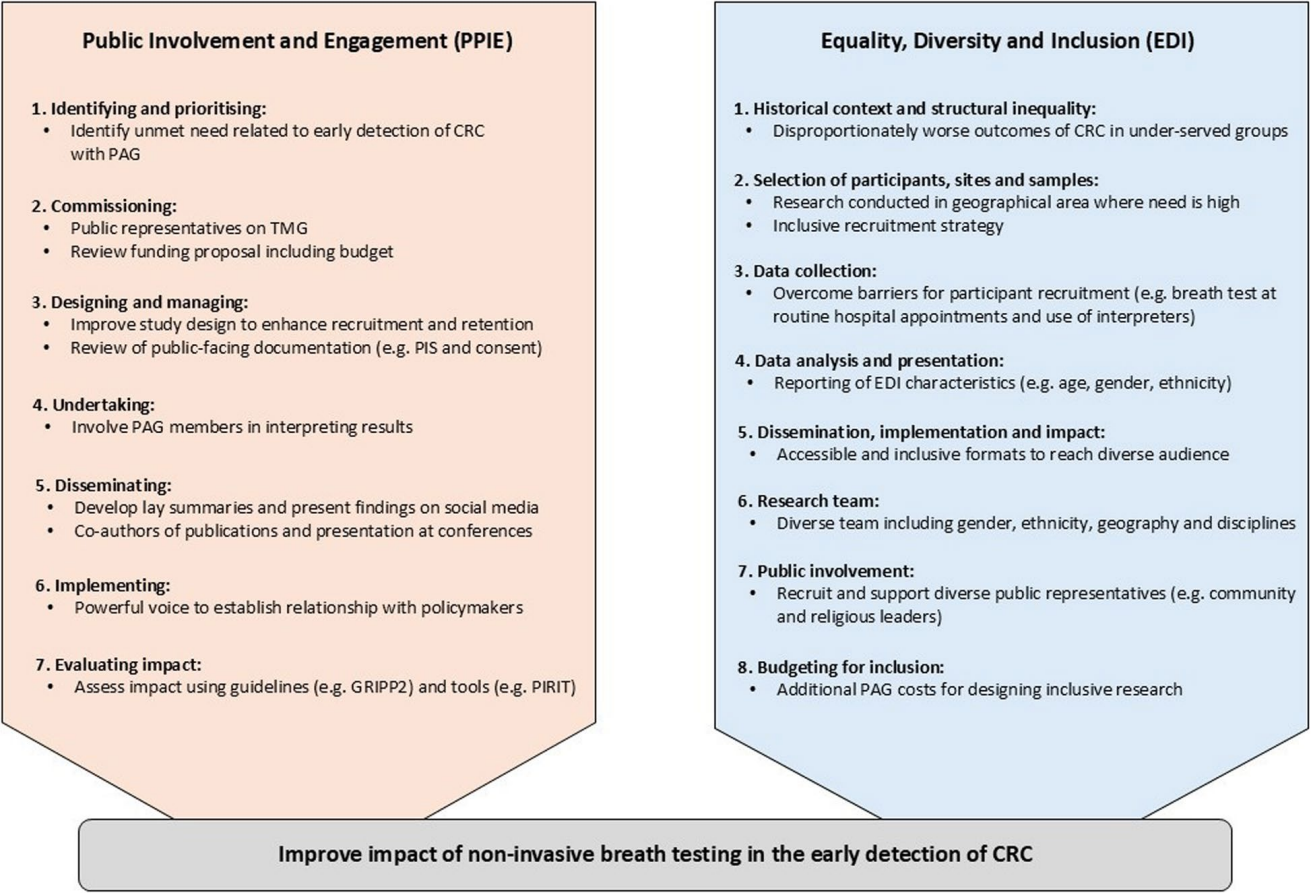
Integration of patient and public involvement (PPIE) and equality, diversity and inclusion (EDI) strategy in the COBRA2 study, based on the different stages of the research cycle [37] and the NIHR Research Design Service EDI toolkit [38]. Abbreviations: CRC, colorectal cancer; GRIPP2, guidance for reporting of patient and public involvement; PAG, public advisory group; PIRIT, Public Involvement in Research Impact Toolkit; PIS, participant information sheet; TMG, trial management group

Patient and public involvement and engagement (PPIE) will be embedded throughout all stages of the COBRA2 study (Fig. 2), including study design, data collection, analysis, dissemination and evaluation [37]. Our PPIE strategy is being led by two patient and public representatives [PW and JH], who sit on the trial management group. We have also formed a diverse public advisory group (PAG) with an additional six members, all of whom have a direct or indirect experience of CRC, to meet regularly and guide the study. The PAG has helped identify unmet needs, refine the participant inclusion criteria, and improve study recruitment pathways. In addition, we will hold focus group meetings with charities, such as Bowel Research UK and Bowel Cancer UK, in order to gain additional perspectives on the acceptability and accessibility of a non-invasive breath test in the detection of CRC.

We will also adopt an equality, diversity and inclusion (EDI) strategy throughout the COBRA2 study. We will be recruiting participants from several UK hospitals to ensure we capture a diverse patient population. The team will draw upon the INCLUDE Ethnicity Framework [39] and the Trial Forge Guidance 3 [40] to identify potential challenges and solutions related to ethnic group participation. For example, we will engage in outreach activities with under-served groups in research, through community events and workshops, to build trust in diagnostic testing. Demographic representation analysis will also be performed to assess participant diversity across age, gender, ethnicity and geographical locations. We will ensure our study materials are clear, accessible, and offered in various formats including paper and digital. If participants are unable to speak English, available NHS translation resources will be used. The PAG will review recruitment numbers to ensure data balance and to address any encountered EDI issues. The PAG will also co-design inclusive and accessible project-related communications and dissemination material (e.g. leaflets, videos, social media).

We will formally assess the impact of PPIE involvement through the Guidance for Reporting Involvement of Patients and the Public (GRIPP2) checklist [41] and the Public Involvement in Research Impact Toolkit [42], (co-designed by our patient and public representative, JH), to reflect on how well we are meeting our aims and identify areas we can improve on. We will also use resources, such as the NIHR Research Design Service EDI toolkit [38] and the Health Inequalities Assessment Toolkit [43], to ensure we carry out EDI in a meaningful, equitable, and sustainable manner throughout the study.

### Ethics and dissemination strategy

The study has received ethical approval from the East of England-Essex Committee and from the Health Research Authority (Ref: 17/EE/0112). Results of this study will be published in open-access peer-reviewed journals, and disseminated through national and international conference presentations. In addition, lay summaries will be shared on our website [44], social media platforms, charities, PPIE forums and community networks, to raise awareness amongst patients and the wider public.

## Discussion

The COBRA2 study aims to develop and validate the CPM for the detection of CRC based on a breath test. The narrow validation will ensure sufficient precision in estimating diagnostic accuracy, discrimination, and calibration whilst maintaining feasibility. The case–control design of symptomatic and confirmed CRC patients has been chosen for COBRA2 to efficiently establish a CRC detection model with a robust reference standard for breath and an enriched cancer population. A large-scale triple-blind broad validation study (COBRA3) will follow in patients where the diagnosis is unknown. Persons collecting breath, carrying out VOC analysis and classifying into cancer/no-cancer will all be unaware of diagnosis. The test will be re-calibrated to reflect CRC prevalence in the broad validation population. A detailed economic analysis will also be performed to fully assess different decision rules from a patient and policy viewpoint.

## Conclusions

In summary, the breath test has the potential to be used by primary care clinicians as a first-line investigation to triage patients presenting with lower GI symptoms and therefore identify high-risk patients who should be referred for colonoscopy. Earlier detection of CRC will allow more patients to receive curative treatment that has a higher chance of success, thereby improving long-term survival and quality of life. The breath test should also ultimately lead to a more efficient and cost-effective service. Furthermore, the developed model has the potential to be applied to different GI cancer types, enabling the integration of data from several clinical studies with aligned methodology, and the development of a platform technology for early detection of multiple GI cancers, such as oesophageal, gastric and pancreatic cancers.

## Supplementary Information

Supplementary Material 1.

## Data Availability

No datasets were generated or analysed during the current study.

## Abbreviations

AUROC: Area under the receiver operating characteristic
CI: Confidence interval
CPM: Clinical prediction model
CRC: Colorectal cancer
DNN: Deep neural network
EDI: Equality, diversity and inclusion
FIT: Faecal immunochemical test
GC-MS: Gas chromatography-mass spectrometry
GDPR: General data protection regulation
GRIPP2: Guidance for reporting involvement of patients and the public
LASSO: Least absolute shrinkage and selection operator
NICE: National Institute for Health and Care Excellence
PPIE: Patient and public involvement and engagement
QC: Quality control
TD: Thermal desorption
TOF: Time-of-flight
VOC: Volatile organic compound
UK: United Kingdom
