# Supplementary material for: Non-invasive breath testing to detect colorectal cancer: protocol for a multicentre, case–control development and validation study (COBRA2 study)
Source: BMC Cancer. 2025 Jul 29;25:1230. doi: 10.1186/s12885-025-14520-2 (PMC12309184; doi:10.1186/s12885-025-14520-2)
Supplement: Supplementary file 1 — Supplementary Material 1. [file 12885_2025_14520_MOESM1_ESM.docx]

**Supplementary material**

| **Variable category** | **Characteristic** |
| --- | --- |
| **Demographics and lifestyle** | - Age - Height (m) - Weight (kg) - Body mass index (kg/m^2^) - Time of last oral intake of food - Time of last oral intake of fluid - Time of last alcohol consumption - Time of last smoke or vape - Gender – Male/Female/Other (please specify) - Ethnic origin – Arab/Asian or Asian British – Bangladesh/Asian or Asian British – Indian/Asian or Asian British – Pakistani/Chinese/Mixed – Asian and White/Mixed – Black African and White/Mixed – Black Caribbean and White/Black or Black British – African/Black or Black British – Caribbean/Gypsy or traveller/White/Other (please specify) - Smoking – Current/Ex-smoker/Never - Number of cigarettes per day if applicable - Alcohol – Current/Ex-smoker/Never - Number of units per week if applicable |
| **Symptoms/reason for referral** | - Change in bowel habit/Diarrhoea/Constipation/Black stool/Rectal bleeding/Weight loss/Abdominal pain/Reduced appetite/Passing mucus/Pain on defecation/Incomplete emptying/Abdominal mass/Rectal mass/Iron deficiency anaemia/Other (please specify)/None - Duration of symptoms (weeks) |
| **Faecal immunochemical test (FIT) result** | - Yes/No - Positive/Negative/Not available/Not performed - Specific value (µg Hb/g) |
| **Past medical history** | - Antibiotics in last 8 weeks/Recent infection in last 8 weeks/Previous cancer in last 5 years/Previous known polyps/Recent bowel surgery/previous colorectal cancer resection/Previous chemotherapy or radiotherapy or immunotherapy for colorectal cancer - Ulcerative colitis/Crohn’s disease/Other IBD/Coeliac disease/Barrett’s oesophagus/Diabetes/Asthma/COPD or emphysema/Other lung disease/Hypertension/Heart disease/Kidney disease/Liver disease/Other (please specify)/None - Previous abdominal surgery - Type of surgery if applicable – Appendicectomy/Cholecystectomy/Hernia Repair/Colectomy/Gastrectomy/Laparotomy/Liver resection/Pancreatic surgery/Splenectomy/Other |
| **Concomitant medications** | - Omeprazole or Lansoprazole/Ranitidine/Aspirin or ibuprofen/Clopidogrel/Anticoagulation/Immunosuppressants including steroids/Laxatives/Antibiotics/Other (please specify)/None |
| **Family history of colorectal cancer in first degree relative (FDR, youngest if multiple)** | - Yes FDR < 50 years old/Yes FDR > 50 years old/No |
| **Breath sampling details** | - Study ID number - Name of participating centre - Date and time of breath sampling - Location of breath sampling – Surgery pre-assessment/Outpatient clinic/Colonoscopy/Theatres - Breath collection device number (breath 1/breath 2/environment) - Breath collection device file number (breath 1/breath 2/environment) - Thermal desorption tube number (breath 1/breath 2/environment) |
| **Control group** | - Colonoscopy overall findings – Normal/Polyp(s)/Diverticular disease/IBD (UC/Crohn’s/indeterminate)/Infectious colitis including *C. difficile*/Colitis of uncertain type/Haemorrhoid/Stricture/Fistula/Other/Unsure - Number of lesions if applicable - Site of lesion – Anus/Ascending colon/Caecum/Descending colon/Hepatic flexure/Ileocaecal valve/Rectum/Sigmoid/Splenic flexure/Terminal ileum/Transverse colon - Size of lesion (mm) - Type of polyp – Stalked/Raised (sessile)/Flat/Depressed - Likely cancerous – Yes/No - Histology (free text) |
| **Cancer group** | - Number of lesions - Site of lesion – Anus/Ascending colon/Caecum/Descending colon/Hepatic flexure/Ileocaecal valve/Rectum/Sigmoid/Splenic flexure/Terminal ileum/Transverse colon - Size of lesion (mm) - TNM staging (TX/T0/T1/T2/T3/T4; NX/N0/N1/N2; M0/M1) - Histology – grade/differentiation (Well/Moderate/Poor) |
| **General comments** | - Free text |

**Table S1.** Participant data collection for the COBRA2 study.

**Abbreviations:** COPD, chronic obstructive pulmonary disease; FDR, first degree relative; IBD, inflammatory bowel disease; TNM, tumour, node, metastasis; UC, ulcerative colitis.
